# Supplementary material for: The Role of Biomarkers and Scores in Describing Urosepsis
Source: Medicina (Kaunas). 2023 Mar 17;59(3):597. doi: 10.3390/medicina59030597 (PMC10059648; doi:10.3390/medicina59030597)
Supplement: Supplementary file 1 [file medicina-59-00597-s001.zip › medicina-2217047-supplementary.pdf]

**Table S1: Pairwise comparison of ROC curves for Deceased group**

| | |
|---|---|
| procalcitonina ~ leucocite | |
| Difference between areas | 0.196 |
| Standard Error [c] | 0.0916 |
| 95% Confidence Interval | 0.0162 to 0.375 |
| z statistic | 2.137 |
| Significance level | P = 0.033 |
| procalcitonina ~ CCI | |
| Difference between areas | 0.0907 |
| Standard Error [c] | 0.0703 |
| 95% Confidence Interval | -0.0472 to 0.229 |
| z statistic | 1.290 |
| Significance level | P = 0.197 |
| procalcitonina ~ SOFA | |
| Difference between areas | 0.121 |
| Standard Error [c] | 0.0536 |
| 95% Confidence Interval | 0.0160 to 0.226 |
| z statistic | 2.258 |
| Significance level | P = 0.024 |
| procalcitonina ~ qSOFA | |
| Difference between areas | 0.131 |
| Standard Error [c] | 0.0595 |
| 95% Confidence Interval | 0.0142 to 0.247 |
| z statistic | 2.199 |
| Significance level | P = 0.028 |
| leucocite ~ CCI | |
| Difference between areas | 0.287 |
| Standard Error [c] | 0.0872 |
| 95% Confidence Interval | 0.116 to 0.457 |
| z statistic | 3.287 |
| Significance level | P = 0.001 |
| leucocite ~ SOFA | |
| Difference between areas | 0.317 |
| Standard Error [c] | 0.0648 |
| 95% Confidence Interval | 0.190 to 0.444 |
| z statistic | 4.888 |
| Significance level | P < 0.001 |
| leucocite ~ qSOFA | |
| Difference between areas | 0.327 |
| Standard Error [c] | 0.0635 |
| 95% Confidence Interval | 0.202 to 0.451 |
| z statistic | 5.142 |
| Significance level | P < 0.001 |
| CCI ~ SOFA | |
| Difference between areas | 0.0304 |
| Standard Error [c] | 0.0482 |
| 95% Confidence Interval | -0.0640 to 0.125 |

| | |
|---|---|
| z statistic | 0.631 |
| Significance level | P = 0.528 |
| CCI ~ qSOFA | |
| Difference between areas | 0.0401 |
| Standard Error [c] | 0.0533 |
| 95% Confidence Interval | -0.0643 to 0.145 |
| z statistic | 0.753 |
| Significance level | P = 0.451 |
| SOFA ~ qSOFA | |
| Difference between areas | 0.00971 |
| Standard Error [c] | 0.0268 |
| 95% Confidence Interval | -0.0427 to 0.0622 |
| z statistic | 0.363 |
| Significance level | P = 0.717 |

**Table S2: Pairwise comparison of ROC curves for SIRS group**

| procalcitonina ~ leucocite | |
|---|---|
| Difference between areas | 0.418 |
| Standard Error [c] | 0.0508 |
| 95% Confidence Interval | 0.319 to 0.518 |
| z statistic | 8.231 |
| Significance level | P < 0.001 |
| procalcitonina ~ SOFA | |
| Difference between areas | 0.0474 |
| Standard Error [c] | 0.0293 |
| 95% Confidence Interval | -0.00998 to 0.105 |
| z statistic | 1.619 |
| Significance level | P = 0.105 |
| procalcitonina ~ qSOFA | |
| Difference between areas | 0.0922 |
| Standard Error [c] | 0.0288 |
| 95% Confidence Interval | 0.0358 to 0.149 |
| z statistic | 3.201 |
| Significance level | P = 0.001 |
| leucocite ~ SOFA | |
| Difference between areas | 0.371 |
| Standard Error [c] | 0.0497 |
| 95% Confidence Interval | 0.274 to 0.469 |
| z statistic | 7.460 |
| Significance level | P < 0.001 |
| leucocite ~ qSOFA | |
| Difference between areas | 0.326 |
| Standard Error [c] | 0.0513 |
| 95% Confidence Interval | 0.226 to 0.427 |
| z statistic | 6.365 |
| Significance level | P < 0.001 |
| SOFA ~ qSOFA | |
| Difference between areas | 0.0448 |
| Standard Error [c] | 0.0264 |
| 95% Confidence Interval | -0.00704 to 0.0966 |

| | |
|---|---|
| z statistic | 1.694 |
| Significance level | P = 0.090 |

**Table S3: Pairwise comparison of ROC curves for Sepsis group**

| procalcitonina ~ leucocite | |
|---|---|
| Difference between areas | 0.0258 |
| Standard Error [c] | 0.0670 |
| 95% Confidence Interval | -0.105 to 0.157 |
| z statistic | 0.385 |
| Significance level | P = 0.701 |
| **procalcitonina ~ SOFA** | |
| Difference between areas | 0.0795 |
| Standard Error [c] | 0.0885 |
| 95% Confidence Interval | -0.0940 to 0.253 |
| z statistic | 0.898 |
| Significance level | P = 0.369 |
| **procalcitonina ~ qSOFA** | |
| Difference between areas | 0.0509 |
| Standard Error [c] | 0.0848 |
| 95% Confidence Interval | -0.115 to 0.217 |
| z statistic | 0.600 |
| Significance level | P = 0.548 |
| **leucocite ~ SOFA** | |
| Difference between areas | 0.0537 |
| Standard Error [c] | 0.0587 |
| 95% Confidence Interval | -0.0613 to 0.169 |
| z statistic | 0.916 |
| Significance level | P = 0.360 |
| **leucocite ~ qSOFA** | |
| Difference between areas | 0.0251 |
| Standard Error [c] | 0.0570 |
| 95% Confidence Interval | -0.0866 to 0.137 |
| **SOFA ~ qSOFA** | |
| Difference between areas | 0.0286 |
| Standard Error [c] | 0.0230 |
| 95% Confidence Interval | -0.0164 to 0.0736 |
| z statistic | 1.245 |
| Significance level | P = 0.213 |

**Table S4: Pairwise comparison of ROC curves for Septic Shock group**

| procalcitonina ~ leucocite | |
|---|---|
| Difference between areas | 0.234 |
| Standard Error [c] | 0.0685 |

| | |
|---|---|
| 95% Confidence Interval | 0.0992 to 0.368 |
| z statistic | 3.407 |
| Significance level | P = 0.001 |
| procalcitonina ~ SOFA | |
| Difference between areas | 0.0985 |
| Standard Error [c] | 0.0381 |
| 95% Confidence Interval | 0.0239 to 0.173 |
| z statistic | 2.587 |
| Significance level | P = 0.010 |
| procalcitonina ~ qSOFA | |
| Difference between areas | 0.0894 |
| Standard Error [c] | 0.0398 |
| 95% Confidence Interval | 0.0114 to 0.167 |
| z statistic | 2.246 |
| Significance level | P = 0.025 |
| leucocite ~ SOFA | |
| Difference between areas | 0.332 |
| Standard Error [c] | 0.0589 |
| 95% Confidence Interval | 0.216 to 0.448 |
| z statistic | 5.633 |
| Significance level | P < 0.001 |
| leucocite ~ qSOFA | |
| Difference between areas | 0.323 |
| Standard Error [c] | 0.0573 |
| 95% Confidence Interval | 0.211 to 0.435 |
| z statistic | 5.640 |
| Significance level | P < 0.001 |
| SOFA ~ qSOFA | |
| Difference between areas | 0.00909 |
| Standard Error [c] | 0.0160 |
| 95% Confidence Interval | -0.0222 to 0.0404 |
| z statistic | 0.569 |
| Significance level | P = 0.569 |

## Table S5: Spearmans' rho correlation between all variables

| | | | varsta | procalcitonina | leucocite | SOFA | qSOFA | CCI | zileATI | Zile spitalizare |
|---|---|---|---|---|---|---|---|---|---|---|
| Spearman's rho | varsta | Correlation Coefficient | 1.000 | 0.251** | -0.153* | 0.392** | 0.291** | 0.688** | 0.102 | 0.164* |
| | | Sig. (2-tailed) | . | 0.001 | 0.045 | 0.000 | 0.000 | 0.000 | 0.179 | 0.031 |
| | | N | 174 | 173 | 174 | 174 | 174 | 174 | 174 | 174 |
| | procalcitonina | Correlation Coefficient | 0.251** | 1.000 | 0.086 | 0.707** | 0.667** | 0.351** | 0.433** | 0.392** |
| | | Sig. (2-tailed) | 0.001 | . | 0.259 | 0.000 | 0.000 | 0.000 | 0.000 | 0.000 |
| | | N | 173 | 173 | 173 | 173 | 173 | 173 | 173 | 173 |
| | leucocite | Correlation Coefficient | -0.153* | 0.086 | 1.000 | 0.157* | 0.153* | -0.003 | 0.073 | -0.027 |
| | | Sig. (2-tailed) | 0.045 | 0.259 | . | 0.039 | 0.044 | 0.968 | 0.337 | 0.726 |
| | | N | 174 | 173 | 174 | 174 | 174 | 174 | 174 | 174 |
| | SOFA | Correlation Coefficient | 0.392** | 0.707** | 0.157* | 1.000 | 0.834** | 0.500** | 0.500** | 0.343** |
| | | Sig. (2-tailed) | 0.000 | 0.000 | 0.039 | . | 0.000 | 0.000 | 0.000 | 0.000 |
| | | N | 174 | 173 | 174 | 174 | 174 | 174 | 174 | 174 |
| | qSOFA | Correlation Coefficient | 0.291** | 0.667** | 0.153* | 0.834** | 1.000 | 0.418** | 0.541** | 0.340** |
| | | Sig. (2-tailed) | 0.000 | 0.000 | 0.044 | 0.000 | . | 0.000 | 0.000 | 0.000 |
| | | N | 174 | 173 | 174 | 174 | 174 | 174 | 174 | 174 |
| | CCI | Correlation Coefficient | 0.688** | 0.351** | -0.003 | 0.500** | 0.418** | 1.000 | 0.218** | 0.125 |
| | | Sig. (2-tailed) | 0.000 | 0.000 | 0.968 | 0.000 | 0.000 | . | 0.004 | 0.099 |
| | | N | 174 | 173 | 174 | 174 | 174 | 174 | 174 | 174 |
| | zileATI | Correlation Coefficient | 0.102 | 0.433** | 0.073 | 0.500** | 0.541** | 0.218** | 1.000 | 0.362** |
| | | Sig. (2-tailed) | 0.179 | 0.000 | 0.337 | 0.000 | 0.000 | 0.004 | . | 0.000 |
| | | N | 174 | 173 | 174 | 174 | 174 | 174 | 174 | 174 |
| | zilespitalizare | Correlation Coefficient | 0.164* | 0.392** | -0.027 | .343** | .340** | 0.125 | 0.362** | 1.000 |
| | | Sig. (2-tailed) | 0.031 | 0.000 | 0.726 | 0.000 | 0.000 | 0.099 | 0.000 | . |
| | | N | 174 | 173 | 174 | 174 | 174 | 174 | 174 | 174 |
